# Angiogenic Effects of Collagen/Mesoporous Nanoparticle Composite Scaffold Delivering VEGF_165_

**DOI:** 10.1155/2016/9676934

**Published:** 2016-09-05

**Authors:** Joong-Hyun Kim, Tae-Hyun Kim, Min Sil Kang, Hae-Won Kim

**Affiliations:** ^1^Institute of Tissue Regeneration Engineering (ITREN), Dankook University, Cheonan, Republic of Korea; ^2^Department of Nanobiomedical Science and BK21 PLUS NBM Global Research Center for Regenerative Medicine, Dankook University, Cheonan, Republic of Korea; ^3^Department of Biomaterials Science, College of Dentistry, Dankook University, Cheonan, Republic of Korea

## Abstract

Vascularization is a key issue for the success of tissue engineering to repair damaged tissue. In this study, we report a composite scaffold delivering angiogenic factor for this purpose. Vascular endothelial growth factor (VEGF) was loaded on mesoporous silica nanoparticle (MSN), which was then incorporated within a type I collagen sponge, to produce collagen/MSN/VEGF (CMV) scaffold. The CMV composite scaffold could release VEGF sustainably over the test period of 28 days. The release of VEGF improved the cell proliferation. Moreover, the* in vivo* angiogenesis of the scaffold, as studied by the chick chorioallantoic membrane (CAM) model, showed that the VEGF-releasing scaffold induced significantly increased number of blood vessel complexes when compared with VEGF-free scaffold. The composite scaffold showed good biocompatibility, as examined in rat subcutaneous tissue. These results demonstrate that the CMV scaffold with VEGF-releasing capacity can be potentially used to stimulate angiogenesis and tissue repair.

## 1. Introduction

Over the past decade, regenerative therapy has been a key issue in the repair of tissues [1, 2]. It requires a well-orchestrated integration of biological events but is often impaired due to pathophysiological situations, including inflammation, fibrosis, or large size of defects [3]. Many scaffolding systems have been developed to recruit and populate cells and to generate microvascular network for sufficient oxygen and nutrients supply [4, 5]. Furthermore, signaling molecules those critically involved in the tissue regenerative process have also been combined with the scaffolds. Because of short half-life in physiological conditions, the signaling molecules need to be secured well within scaffolds and then be released in a sustained manner [6, 7].

Here we design scaffolds that can incorporate signaling molecules safely and then release them sustainably. Collagen type I was prepared with a foam type to serve as the main scaffold matrix. Collagen type I has been widely studied as tissue engineering scaffolds [8]. Mainly the fibrillar form of collagen with sufficient self-assembly process could improve the physical and biochemical stability [9–11]. As the signaling molecule, we focused on angiogenic factors. Early vascular response for angiogenesis is required to repair damaged tissue effectively [1, 12]. Among the angiogenic factors commercially available over 20 [13, 14], we used 165-amino-acid form of vascular endothelial growth factor (VEGF_165_). VEGF, as a potent inducer of angiogenesis [1], serves as a good candidate for delivery as part of engineering several organs, in a number of different systems [15–21]. In particular, VEGF_165_ was incorporated with nanospherical carrier, mesoporous silica nanoparticle (MSN). MSN thus serves as the reservoir of VEGF_165_; in this way, the growth factor can be secured safely and then be released sustainably. In fact, MSN has been studied to deliver many biological molecules including chemical drugs, enzymes, genes, and proteins [9, 22, 23]. Because of the high mesoporosity of MSN, those molecules can be loaded effectively [24]. MSN is also known to have good cell and tissue compatibility [6].

Although the MSN delivery system has been extensively studied, its combination with scaffold systems to deliver signaling molecules has been limitedly studied. Our group has recently demonstrated that the MSN incorporating growth factors could release acidic fibroblast growth factor in a highly sustained manner when combined with collagen scaffolds [6]. In this study, the collagen/MSN/VEGF (CMV) sponge examined the capacity to stimulate angiogenesis using chick chorioallantoic membrane (CAM) model. The VEGF release and the biocompatibility of the composite scaffold were also investigated.

## 2. Materials and Methods

### 2.1. Preparation of Composite Foam Scaffolds Loading VEGF

All the chemicals were purchased from Sigma-Aldrich and used as received. The preparation of MSNs was based on the method described elsewhere [6]. In brief, 5 g of cetyltrimethylammonium bromide was dissolved in a mixture of 200 mL of ethanol, 22 mL of DW, and 8 mL of 30% NH_4_OH and was followed by the addition of 0.2 mL of tetraethyl orthosilicate (TEOS). The homogeneous suspension obtained, and solid nanoparticles were recovered by centrifugation. The nanoparticles were washed and dried overnight at 70°C. After this, the powders were washed with 1% ammonium nitrate solution at 60°C to completely remove the surfactant. Ultrastructural features of the nanoparticle were observed by transmission electron microscopy (TEM; JEM-3010, JEOL, Tokyo, Japan), and the particle size was analyzed based on the TEM images.

**Figure 1:**
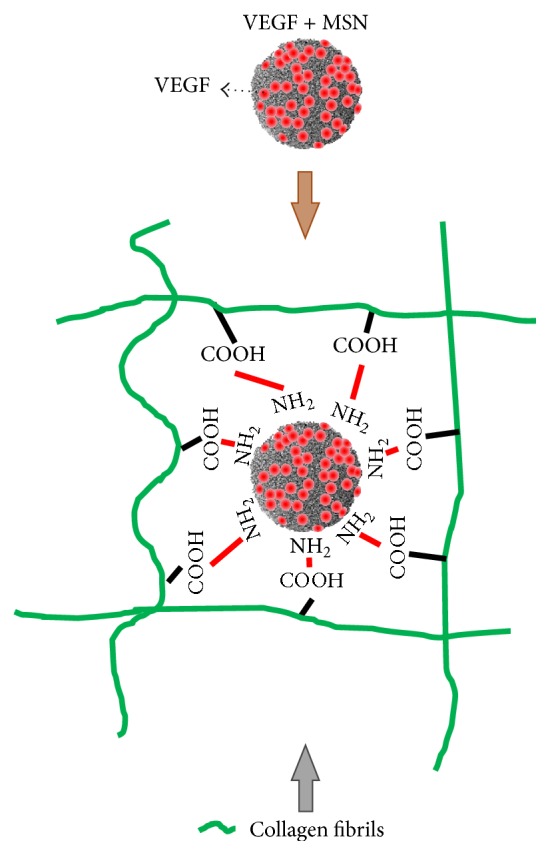
Schematic illustration showing the design of collagen-MSN composite scaffold that delivers VEGF.

In order to incorporate growth factor into the sponge type scaffold, 250 ng recombinant human vascular endothelial growth factor 165 (rhVEGF_165_) (#293-VE-050/CF, R&D system) was loaded in 1 mg MSN and incubated 4 hours at the room temperature. The schematic of the strategy for sponge type scaffold showing the binding VEGF incorporated MSN with the type I collagen fiber is shown in Figure 1. Collagen/MSN (CM) scaffolds were fabricated using a process developed within our laboratory. Briefly, 30 mg of type I rat tail collagen solution (#354236, Corning) was added to 40 mL of 10x PBS and incubated 30 mins at 37°C to reconstituted cross-linked collagen fibrils. The 1 mg MSN with/without rhVEGF_165_ was gently impregnated within obtained 3 mg fibrous collagen suspension. After that the mixture was homogenized and centrifuged at 10,000 rpm for 20 min. After the supernatant was removed, the obtained collagen gels were immersed in distilled water to remove residual NaCl at room temperature. After this step, the resulting gels were moved into the 5 mm diameter mold to give shape and followed by freeze-drying. After 12 h, the sponge type was removed from the mold and ready for use. The morphology of the prepared scaffolds was carried out by scanning electron microscopy (SEM, JSM-6510, JEOL, Japan). The freeze-dried samples were transferred into liquid nitrogen for 15 min and mechanically fractured at the center to see the interconnection between collagen and MSN. The samples were placed on a metallic sample holder, and sputtered with gold to observe the cross sections.

### 2.2. *In Vitro* Growth Factor Release Test

To examine release profile, the four scaffolds per each group were immersed in 1 mL PBS and incubated at 37°C for up to 28 days. At given time points, 1 mL aliquot of the supernatant was collected and replaced by fresh PBS. The amount of rhVEGF_165_ released from the aliquots was measured using human VEGF ELISA development kit (#900-K10, Peprotech) according to the manufacturer's instructions. Briefly, 100 *μ*L capture antibody was added to each well of a 96-well ELISA plate and incubated overnight at room temperature. Subsequently, the plate was washed three times, and the nonspecific binding sites in the wells were blocked by adding 300 *μ*L of blocking buffer. After washing, VEGF samples and the serially diluted standard protein were added to the wells and incubated for 2 h. The plate was washed and 100 *μ*L of detection antibody was added to each well. After 30 min, the plate was washed and the assay was developed by the addition of 2,2′-azino-bis(3-ethylbenzthiazoline-6-sulphonic acid) reagent (0.03%, w/v, Sigma-Aldrich). The absorbance was measured using an iMark microplate reader (BioRad, USA).

### 2.3. Rat Bone Marrow Derived Mesenchymal Stem Cell Culture

The bioactivity of the scaffolds loaded with rhVEGF_165_ was studied using primarily cultured bone marrow derived rat mesenchymal stem cells (rMSCs), in order to study the effects of the scaffolds on mesenchymal stem cells (MSCs) proliferation* in vitro*. Animal cell primary culture was performed after the approval by Dankook University Institutional Animal Care and Use Committee, Republic of Korea. Rat MSCs were isolated from 5-week-old male Sprague-Dawley (SD) rat (Daehan Biolink Co., Ltd., Chungbuk, Korea). Briefly, femurs and tibias of SD rat were harvested and the soft tissues were removed. The proximal end and the distal end of each femur and tibia were removed using sterile scissors. The bone marrow area was flushed, and bone marrow cells were collected. Flushed marrow was digested with 0.05% collagenase type I solution for 30 min in an incubator under a humidified atmosphere of 5% CO_2_ in air at 37°C. Single cell suspension was washed and resuspended in fresh primary culture medium which consisting of *α*-modified minimal essential medium (*α*-MEM) supplemented with 10% fetal bovine serum (FBS) and containing 100 U/mL penicillin and 100 mg/mL streptomycin. The cultures were rinsed for removal of nonadherent cells and expanded. The medium was refreshed every 2-3 days throughout the studies; confluent cells were detached by trypsinization (0.05% trypsin-EDTA). The cells at 3 passages were used for further experiments.

### 2.4. Indirect Cell Bioactivity

*In vitro* proliferation of the primarily cultured rMSCs from released growth factor was evaluated on the first, third, fifth, and seventh days. For the indirect test, 5 × 10^3^ rMSCs were plated onto each well of 24-well plates, and after 2 hours of cell adhesion, the scaffold samples were transferred into insert. The cell culture medium was refreshed every 3.5 days. The samples were incubated at 5% CO_2_ in air at 37°C, and every 2 days, the proliferation effect was measured. Quantification of the cell proliferation was assessed by cell counting kit-8 (CCK, Dojindo), according to the manufacturer's instructions. Briefly, the cultured cells were washed and incubated with 10% CCK reagent included media for 2 hours in the incubator. An aliquot from each well (100 *μ*L) was transferred to a 96-well plate, and absorbance of each well was measured at 450 nm using an iMark microplate reader. Four samples in each group were used and data were averaged from duplicate tests. Intensity of experimental samples was then interpolated to determine the cell proliferation (*n* = 4). In order to examine the cell attachment and spreading, the seeded cells from the proliferation test were imaged using phase contrast microscope.

### 2.5. CAM Assay

Once the bioactivity of the eluting scaffolds was confirmed* in vitro*, scaffolds with a diameter of 5 mm were implanted into the window generated on egg in order to assess the ability of CMV scaffolds to promote angiogenesis. Briefly, eggs of chickens were incubated at 37.8°C and 70–80% humidity. On the 3rd day of development, the eggs were punctured and 3 mL of the liquid was removed. The egg was sealed and returned to the incubator until day 7. The scaffolds were placed in contact with the CAM of 7-day-old embryos and incubated four more days. After sacrificing the embryos, the CAM with the implanted scaffolds were fixated in 10% formalin and processed for further visualization. Images were collected with a stereomicroscope (10x magnification) and analyzed by an AngioQuant Standalone software (MATLAB, Inc. Tampere, Finland). For comparative purposes, a second group received growth factor unloaded CM scaffolds, while a third group received no scaffold and these eggs served as a negative control (*n* = 8).

### 2.6. Animal Implantation Surgery

As the animal models, rat subcutaneous model was used to investigate the tissue compatibility of the experimental samples. All animals were treated and all surgical procedures were conducted protocols approved in accordance with the Animal Care and Use Committee, Dankook University, Cheonan, Korea. Twelve-week-old male SD rats (weighing approximately 350 g) were divided into two groups of two rats each and used in the animal tests for 2 weeks. The experimental groups for this scaffold system include CM and CMV. Each rat had been anesthetized with xylazine hydrochloride 10 mg/kg and ketamine hydrochloride 80 mg/kg by intramuscular injection. The skin of dorsal area was shaved and the operative area was prepared with serial using antiseptic surgical scrub, povidone-iodine, and 70% ethanol, prior to draping. A 2 cm longitudinal skin incision was made, and two small pouches were subcutaneously prepared on the lateral side from the incision. Scaffolds were implanted into the prepared pouch, and skin was subsequently closed with 4-0 nonabsorbable monofilament sutures (Prolene®, Ethicon, Germany). After the implantation, the rats were fed with sterile food and water* ad libitum* and housed individually in sterilized cages in a barrier system with a relative temperature (20–24°C) and humidity (30–70%) following a 12 h light/dark cycle.

### 2.7. Histological Analysis

In addition to CAM assay, qualitative histological examination was performed in order to further assess the biocompatibility of the scaffold. Two weeks after implantation, the animals were sacrificed by CO_2_ asphyxiation and cervical dislocation. The skin of the rats was excised and the scaffolds were individually dissected and harvested. The harvested tissues were immediately fixed with 10% neutralized buffered formalin overnight. After fixation, specimens were dehydrated in a graded series of ethanol solutions, bisected, and embedded in paraffin using standard procedures. The embedded specimens were sectioned (5 *μ*m thickness) along the longitudinal axis of the scaffold from midportion of each sample using a rotary microtome (Leica RM2455) and mounted on poly-l-lysine coated glass slides. Prepared slides were deparaffinized and were stained with hematoxylin and eosin (H&E) and Masson's trichrome (MT). The images of each specimen were visualized under IX71 microscope (Olympus, Tokyo, Japan) equipped with the MetaMorph software (Molecular Devices Corporation, Pennsylvania, USA).

### 2.8. Statistical Analysis

Data are given as the mean and standard deviation (SD). Statistics were carried out by one-way analysis of variance (ANOVA) with a *p* value < 0.05 considered statistically significant.

## 3. Results

### 3.1. Collagen/MSN/VEGF Scaffold

**Figure 2:**
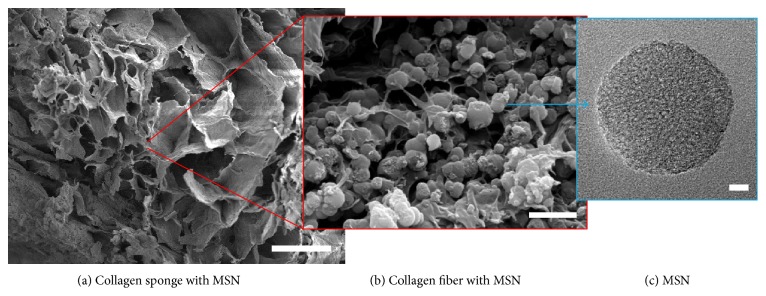
Scanning electron microscopy (SEM) and transmission electron microscopy (TEM) images of the composite scaffolds. Cross-sectional SEM image of the scaffold (bar = 100 *μ*m) (a). Higher magnification SEM image of MSN-distributed collagen (bar = 1 *μ*m) (b). TEM image of MSN (bar = 10 nm) (c).

The morphologies of composite scaffold (CMV) were examined by SEM. A well-interconnected porous structure was developed at low magnification (Figure 2(a)), a typical of freeze-dried scaffolds [4]. The distribution of MSNs within the collagen network was also revealed at high magnification (Figure 2(b)). Average pore size was 654.5 ± 115.4 *μ*m, and the porosity was 84.4 ± 2.3%. The spherical and highly mesoporous structure of the MSN was observed by TEM (Figure 2(c)). The particle size was measured from TEM images. Thirty particles were counted and the mean size with standard deviation was 205 ± 32 nm.

### 3.2. VEGF Release Behavior

**Figure 3:**
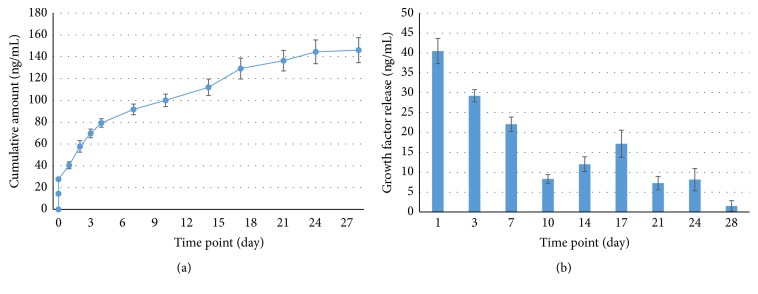
*In vitro* release profile of VEGF from the scaffold. Accumulative (a) and incremental (b) release of VEGF.

The* in vitro* release profile of VEGF from the CMV scaffold was examined by an ELISA quantification assay.  Figure 3(a) shows the cumulative amount of VEGF released from the CMV scaffold. After a short burst release (within few hours), the VEGF release was gradual and continued up to 28 days. The incremental release curve replotted in Figure 3(b) also explained the release behavior with time. The total release amount was approximately 140 ng, which corresponded to 56% of the incorporated VEGF.

### 3.3. *In Vitro* Bioactivity

**Figure 4:**
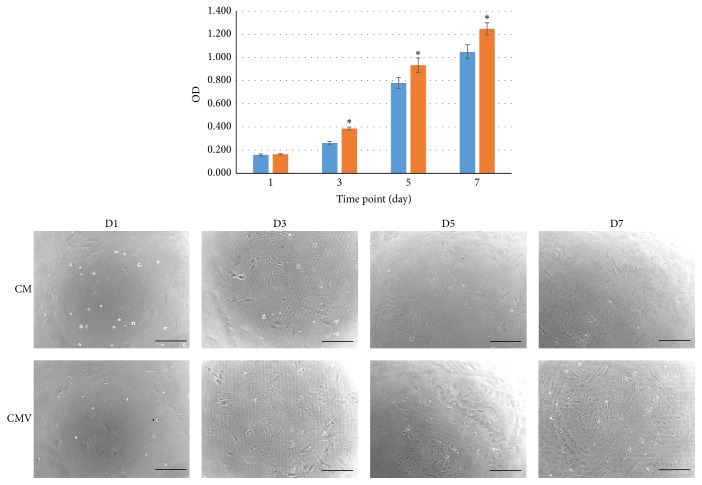
*In vitro* proliferation of rMSCs cultured with scaffolds. The cells were cultured in plates, with the scaffolds placed in transwell insert. The graph represents mean ± SD from 4 replicate samples. Statistically significant difference was observed. Light microscopic images of the cells are also shown (×100, bar = 300 *μ*m, ^*∗*^ *p* < 0.05 compared to CMV).

To determine whether the released VEGF from the CMV scaffold might affect cellular viability and proliferation, the scaffolds were placed in a transwell insert membrane to allow indirect interaction with rMSCs. Cell proliferation level was compared between the scaffolds that are either releasing VEGF or not releasing VEGF, by means of CCK up to 7 days. The results demonstrated that the released VEGF was significantly influencing the cellular growth (Figure 4). The cell images taken by optical microscopy again explained the stimulatory effects of VEGF on the cell proliferation.

### 3.4. Angiogenesis Assessed by CAM Model

**Figure 5:**
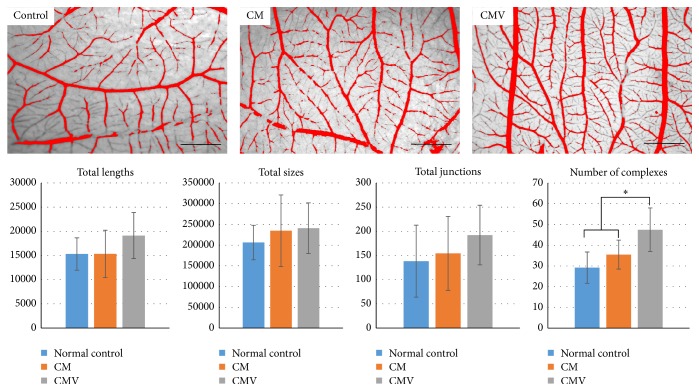
Representative images of blood vessel networks formed in the CAM model after 4 days of sample placement in contact with embryos (×10, bar = 200 *μ*m). Image analysis represents total length, total size, total junction, and number of complexes of blood vessels for control, CM, and CMV group (mean ± standard deviation, ^*∗*^ *p* = 0.00002 compared to CMV).

To evaluate the potential of the CMV scaffold for angiogenesis in CAM assay, the scaffolds were implanted into the egg. Qualitative and quantitative results of CAM assay are represented in Figure 5. After 4 days, increased capillary branches were observed under the stereomicroscopy in CMV group when compared with the other groups (CM or normal egg control group). The number of the newly formed blood vessel complexes was the highest in the CMV, with statistically significant difference, although other parameters (total length, size, and junction) were not statistically significant between the groups.

### 3.5. *In Vivo* Biocompatibility

**Figure 6:**
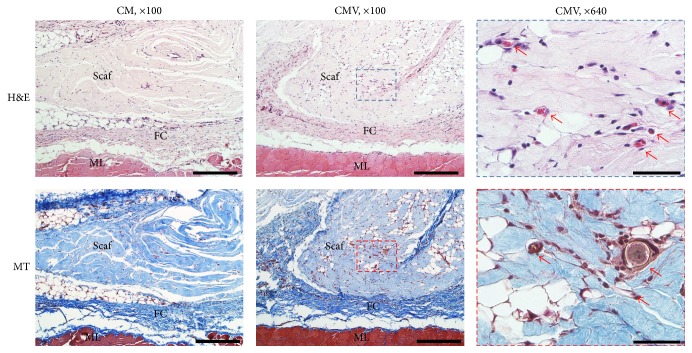
Qualitative histologic analysis of tissue samples after implantation in rat subcutaneous model for 2 weeks. H&E and MT stain showing histological images of the interface between connective tissue and scaffolds (scale bar = 300 *μ*m). Vessels are characterized by the presence of red blood cells within defined lumens lined by endothelial cells at higher magnification (ML: muscle, FC: fibrous capsule, Scaf: scaffold, red arrow: blood vessel, and scale bar = 50 *μ*m).

The final observation of this study was to examine the* in vivo* compatibility of the VEGF-releasing scaffolds (Figure 6). Scaffolds were implanted in a rat subcutaneous tissue, and the tissue histology was examined after 2 weeks. All animals showed normal activity within 1-2 days after operation. The histological examination showed that both scaffolds used were biocompatible. The scaffolds did not show a sufficient level of degradation sign and are compatible within the surrounding dense connective tissue. Macroscopic signs of immune reactions or tissue rejections were absent, and surrounding tissue was in its native form for both groups. Many fibroblastic cells were found around the scaffold. Thin fibrous capsule with neovascularization was also shown around the samples. Of note, the histology results clearly indicated that numerous capillaries were present within scaffold area in animals treated with CMV (at higher magnification).

## 4. Discussion

In the present study, we designed a novel growth factor delivering scaffold to stimulate angiogenesis. When growth factors are delivered, their availability to the local microenvironment is not well controlled or sustained over time, because of their short* in vivo* half-life by the denaturation and enzymatic degradation [1]. To overcome this, the angiogenic growth factor VEGF_165_ was impregnated indirectly within sponge type collagen scaffolds through MSN as the growth factor carrier. MSNs have been widely used as delivery vehicles for drug, genes, and growth factors, thanks to the highly mesoporous structure and the tunability of mesopore properties [1, 6, 9]. Electron microscopic images revealed the production of mesoporous nanoparticles well impregnated within the collagen fibrillar macroporous sponge scaffold. This feature of nanocomposite scaffolds is considered to provide channels for oxygen and nutrients supply while incorporating VEGF within the mesopores [9]. In particular, the current MSN was positively charged by an amine functionalization, which could allow for the effective loading of VEGF through weak charge-charge interaction [6, 9].

VEGF was sustainably released from the MSN-collagen scaffolds. After a burst release within few hours, VEGF release continued over as long as 28 days. This burst effect is due to the loosely bound VEGF possibly prereleased and present in the collagen foam that might occur during the mixing process. In fact, proteins (including growth factors) are known to be released from MSNs for a short time period, mostly in a few weeks. However, such a long-term release kinetics is due to the presence of collagen matrix. As soon as the VEGF is released, it should diffuse out through the macropore channels of collagen foam. Thus the presence of collagen around MSNs, that is, the combination of MSNs with collagen matrix, is considered to play significant role in sustaining the release profile of the growth factor [6]. However, only 56% of VEGF was released from the scaffold, and 44% remained. The remaining VEGF may be strongly entrapped within MSN, because MSN has highly porous structure with positively charged surface. This porous structure provided enough space for growth factors loading, and the positive charge provided strong interaction between growth factor and MSN [6].

Due to the release of VEGF the MSCs proliferation was enhanced significantly, as confirmed by the transwell insert assay. This finding is also in good agreement with the previous study by Ball et al. who proved that VEGF concentration of 10 ng/mL had a significant effect on the MSC growth [25]. In our study, the released VEGF concentration could be maintained well above 20 ng/mL for 7 days, and approximately 7–17 ng/mL thereafter.

The effects of VEGF delivery on the angiogenesis were examined by CAM model, one of the classical assays for studying the effects of angiogenic factors* in vivo* in a quick, semiquantifiable, and efficient way [14]. The image analysis quantified the newly formed vessel complex number through the VEGF-releasing scaffold. The vessel complex number of CMV was ~62.6% higher than that of control group and ~33.7% higher than that of the VEGF-free scaffold. The increased blood vessel complex number in the VEGF-releasing scaffold signifies the formation of denser capillary network, which can provide better gas and essential nutrition exchange for tissue regeneration [14].

The composite scaffolds were implanted in a rat subcutaneous tissue to observe the angiogenic effect with inflammatory response, fibroblast proliferation, and collagen deposition. Two weeks are generally proper to study the angiogenic effect of VEGF at a relatively early phase of tissue repair [6]. The histological appearance of CM was similar to that of CMV scaffolds, except blood vessel formation. There was substantial invasion of cells into both CM and CMV groups, and the fibrous capsules surrounding scaffolds were thin without immune cells. The central region of the scaffolds was fully populated with migrated cells. This effective cellular migration is of special importance in the regeneration processes of damaged tissues [26]. This results may be achieved by increasing scaffold porosity, which may facilitate cell migration and blood vessel ingrowth into the scaffolds [27]. More importantly, the prominent new vascular network formation was observed within the CMV scaffold, which is clearly evident at high magnification. Collagen is known extracellular matrix, which promotes epithelial cell proliferation [28].

Taken all, this study demonstrated the angiogenic effect of the designed scaffolds delivering VEGF_165_, which thus could accelerate wound healing process for tissue regeneration. For further study,* in vivo* study can be performed to confirm the potency of this system as a cell delivery tool for cell therapy to preserve viability of the delivered cells MSCs or other cells for tissue regeneration [28]. And not only single delivery of VEGF but also dual delivery system with other therapeutic biomolecules will be promising to achieve more specified cell and tissue functions like bone formation through angiogenesis [29].

## 5. Conclusions

Here we showed that the MSN-collagen scaffolds could incorporate VEGF_165_ effectively and be delivered sustainably. The VEGF-releasing scaffolds stimulated cell proliferation and enhanced blood vessel formation in CAM model and rat subcutaneous tissue model, suggesting a suitable 3D matrix for tissue regeneration where angiogenesis is important.
